# The Crystal Structure of the Human Co-Chaperone P58^IPK^

**DOI:** 10.1371/journal.pone.0022337

**Published:** 2011-07-25

**Authors:** Maria Svärd, Ekaterina I. Biterova, Jean-Marie Bourhis, Jodie E. Guy

**Affiliations:** Department of Medical Biochemistry and Biophysics, Karolinska Institutet, Stockholm, Sweden; Consejo Superior de Investigaciones Cientificas, Spain

## Abstract

P58^IPK^ is one of the endoplasmic reticulum- (ER-) localised DnaJ (ERdj) proteins which interact with the chaperone BiP, the mammalian ER ortholog of Hsp70, and are thought to contribute to the specificity and regulation of its diverse functions. P58^IPK^, expression of which is upregulated in response to ER stress, has been suggested to act as a co-chaperone, binding un- or misfolded proteins and delivering them to BiP. In order to give further insights into the functions of P58^IPK^, and the regulation of BiP by ERdj proteins, we have determined the crystal structure of human P58^IPK^ to 3.0 Å resolution using a combination of molecular replacement and single wavelength anomalous diffraction. The structure shows the human P58^IPK^ monomer to have a very elongated overall shape. In addition to the conserved J domain, P58^IPK^ contains nine N-terminal tetratricopeptide repeat motifs, divided into three subdomains of three motifs each. The J domain is attached to the C-terminal end via a flexible linker, and the structure shows the conserved Hsp70-binding histidine-proline-aspartate (HPD) motif to be situated on the very edge of the elongated protein, 100 Å from the putative binding site for unfolded protein substrates. The residues that comprise the surface surrounding the HPD motif are highly conserved in P58^IPK^ from other organisms but more varied between the human ERdj proteins, supporting the view that their regulation of different BiP functions is facilitated by differences in BiP-binding.

## Introduction

P58^IPK^ (58-kDa inhibitor of protein kinase) is a member of the endoplasmic reticulum- (ER-) localised DnaJ family of proteins (ERdj proteins) which interact with BiP, an important regulator of the functions of the endoplasmic reticulum (ER) (recently reviewed by Otero et al. [1]). BiP, the mammalian ER ortholog of Hsp70, is involved in folding and maturation of nascent proteins as well as targeting misfolded proteins for degradation and regulating the unfolded protein response. Like other members of the Hsp70 family, BiP interacts with DnaJ proteins (also known as Hsp40s); binding of the J domain to the ATPase domain of BiP enhances its ATPase activity and enables a tighter binding between BiP and its substrate. In the mammalian ER, there are seven BiP-binding J domain proteins (ERdj1-7) described to date. These different ERdj proteins have been suggested to function as co-chaperones, and are thought to contribute to the specificity and regulation of the diverse functions of BiP [1]. The ERdj proteins all contain the J domain, but beyond this they have little or no sequence identity, and all contain very different functional domains in the remainder of their sequence. This structural and functional diversity is key to their emerging roles in regulating the various functions of BiP.

P58^IPK^, also known as ERdj6 or DnaJC3, contains the conserved J domain close to its C-terminus, and a 41 kDa N-terminal domain comprised of nine tetratricopeptide repeat (TPR) motifs. P58^IPK^ has several known interaction partners and, like many members of the ERdj family, it appears to have quite diverse functions in the cell; it was initially identified as a cytosolic inhibitor of PKR (RNA-activated protein kinase) in influenza infection [2]. During the cellular response to viral infection PKR, upon activation by dsRNA, phosphorylates the α subunit of the eukaryotic initiation factor 2 (eIF2α), leading to decreased synthesis of host and viral proteins. In order to prevent these events, the virus recruits P58^IPK^ to inactivate PKR, thereby allowing viral protein synthesis to continue [2]. Binding of P58^IPK^ to PKR is inhibited through interaction with another protein, P52^rIPK^ (52-kDa repressor of the inhibitor of protein kinase) [3].

The transcription of P58^IPK^ was later shown to be induced in response to ER stress [4]. When unfolded proteins accumulate in the ER, specific pathways, collectively termed the unfolded protein response (UPR) (recently reviewed by e.g. Wiseman et al., Ron & Walter, and Zhang & Kaufman [5]–[7]), are evoked. This ultimately leads to reduced import of proteins into the ER and upregulation of genes encoding ER chaperones and components of the ER associated degradation pathway. P58^IPK^ is one of the proteins that are transcriptionally induced during the UPR and is known to bind PERK (PKR-like ER kinase), one of the key regulators of the response, controlling PERK-dependent phosphorylation of eIF2 in the later stages of the response [4].

While P58^IPK^ had already been linked to the UPR by its inhibition of PERK, its direct interaction with BiP came to light after the cellular localisation of the protein was re-examined by Rutkowski and co-workers [8]. P58^IPK^ is now known to contain an N-terminal ER-localisation signal, and to be found primarily in the ER lumen; it is thought that slightly inefficient translocation explains the small fraction of the protein remaining in the cytosol [8]. The roles of P58^IPK^ in the ER lumen and its involvement in the UPR have been the focus of many recent studies. Its confirmed involvement with BiP suggested a co-chaperone function, a role that has been endorsed by recent findings indicating that P58^IPK^ can also make a direct interaction with unfolded protein substrates [9]. A co-chaperone function is further supported by the fact that P58^IPK^ deficient cells show a reduced ability to cope with misfolded proteins [8], as well as the fact that knockout of the gene encoding P58^IPK^ in a mouse model causes a mild diabetic phenotype [10]. Most recently it has been proposed that P58^IPK^ binds unfolded protein substrates, stabilises them, and then subsequently binds BiP and facilitates the transfer of the substrate to the chaperone [9]. While it looks likely that this is the case, many questions remain, not least concerning the hand-over of substrate from P58^IPK^ to BiP. Recently, the first structure of the TPR domain of the mouse P58^IPK^ protein was determined [9]. This identified a putative binding site for unfolded protein, but could not provide any information on the interactions with BiP due to the absence of the J domain. Structural information on the interacting domain of BiP is available [11], but structures of the J domains of the ERdj proteins, and ultimately of BiP-ERdj complexes, will be necessary in order to determine the similarities and differences in their interactions and to understand their roles in regulating the functions of BiP.

In this study we present the structure of human P58^IPK^, including the BiP-binding J domain. The crystal structure reveals P58^IPK^ to be an elongated protein, with the J domain attached to the very end of the TPR domain via a flexible linker. From the structure we can identify the probable binding site for BiP based on the position of the conserved histidine-proline-aspartate (HPD) motif which is necessary for Hsp40-Hsp70 interactions. This motif is positioned on the very edge of the elongated protein and at a distance of 100 Å from the previously suggested site for binding of unfolded protein substrates. The large distance between the two binding sites will have implications for the mechanism by which unfolded protein is passed from the co-chaperone to BiP.

## Results and Discussion

### Purification and crystallisation

Multiple constructs of the mature P58^IPK^, lacking the N-terminal signal recognition sequence, were initially prepared and screened for solubility with varied success. No constructs including the 43 C-terminal amino acid residues, which are predicted to be unstructured, could be expressed in soluble form. The longest soluble construct (residues 35–461) therefore consisted of the TPR domain and the J domain, without these C-terminal residues, and this was used for crystallisation. Extensive screening for crystallisation conditions was performed using protein both with and without the N-terminal His-tag that was included in the expression construct, but crystals were obtained in only a single condition using protein containing the intact His-tag (crystal form 1). Despite extensive optimisation and screening of many crystals at the synchrotron beamline, these native crystals did not diffract beyond 4–5 Å. However, selenomethionine-substituted crystals, intended for phasing purposes, diffracted to a maximum of 3.2 Å and provided the dataset which was used to solve the structure in space group P312 using a combination of molecular replacement and single wavelength anomalous diffraction phasing. Finally, it was found that using this optimised condition to crystallise native protein, after enzymatic cleavage of the His-tag, yielded crystals that looked very similar but diffracted in space-group H32 (crystal form 2). These crystals provided the dataset used to refine the structure at 3.0 Å. The two crystal forms are very closely related. Crystal form 1 (P312) has one chain in the asymmetric unit and crystal form 2 (H32) has three chains. If chain A of crystal form 2 is superimposed on the single chain in crystal form 1, chains B and C are shifted only 2–4 Å and 1–2 Å, respectively, from symmetry-generated chains in the other space-group. The crystals have estimated solvent content of approximately 72% in crystal form 1 and 71% in crystal form 2. Table 1 shows a summary of the statistics for each of the datasets.

**Table 1:** Data collection statistics.

Data set	Crystal form 1, Se-Met	Crystal form 2
Space group	P312	H32
Unit cell (Å)	124.6, 124.6, 92.22	212.3, 212.3, 283.5
Molecules in asymmetric unit	1	3
Resolution (Å)	30.0-3.20 (3.37–3.20)	25.0-3.00 (3.16-3.00)
R_merge_	0.090 (0.602)	0.082 (0.557)
<I>/<σI>	8.9 (2.1)	11.5 (2.2)
Completeness (%)	99.9 (100.0)	96.6 (79.2)
Multiplicity	4.6 (4.5)	4.3 (3.7)
Anomalous completeness (%)	99.3 (99.4)	
Anomalous multiplicity	2.4 (2.3)	
Wilson B-factor (Å^2^)	74.7	67.4

Data in parentheses are for the highest resolution shell.

### Quality of the electron density maps and models

In both structures, the mainchain density for P58^IPK^ is essentially complete and the majority of sidechains are clearly visible. In both crystal forms, the electron density in the TPR domain is of particularly high quality for the moderate resolution, while the density for the J domain is weaker. The linker between the TPR domain and the J domain (particularly residues 402–408) and a loop in the J domain (particularly residues 425–428) show highest flexibility, however even these regions are relatively well resolved in chain A of crystal form 2, which enabled model building. The final models of P58^IPK^ both contain residues 35–455. The refinement statistics are summarised in Table 2. The structure in the two crystal forms is very similar, with root mean square deviation (RMSD) of 0.8 Å (using chain A from crystal form 2). The three chains in the higher resolution structure of crystal form 2 show RMSD of 0.4 Å between A and B, 0.5 Å between A and C and 0.7 Å between B and C. In this crystal form, the density quality is highest, and B-factors lowest, in chain A, while B and C have weaker density. Due to the high density quality of chain A, all analysis is based on this molecule unless stated otherwise. Composite omit maps showing the electron density corresponding to each of the domains and the linker between them in this chain are shown in Figure S1.

**Table 2:** Refinement and model building statistics.

Data set	Crystal form 1, Se-Met	Crystal form 2
***Refinement statistics***		
Reflections in working set	12967	44987
Reflections in test set	677	2416
R-factor/R-free	0.244/0.286	0.243/0.291
Number of protein atoms	3429	10259
Average B-factor (including TLS contribution) (Å^2^)	118.5	107.1
***RMSD values from ideal***		
Bond lengths (Å)	0.012	0.008
Bond angles (°)	1.26	1.04
***Ramachandran plot***		
Favoured regions	95.02%	96.58%
Outliers	0.47%	0.32%

### Overall structure of human P58^IPK^

**Figure 1:**
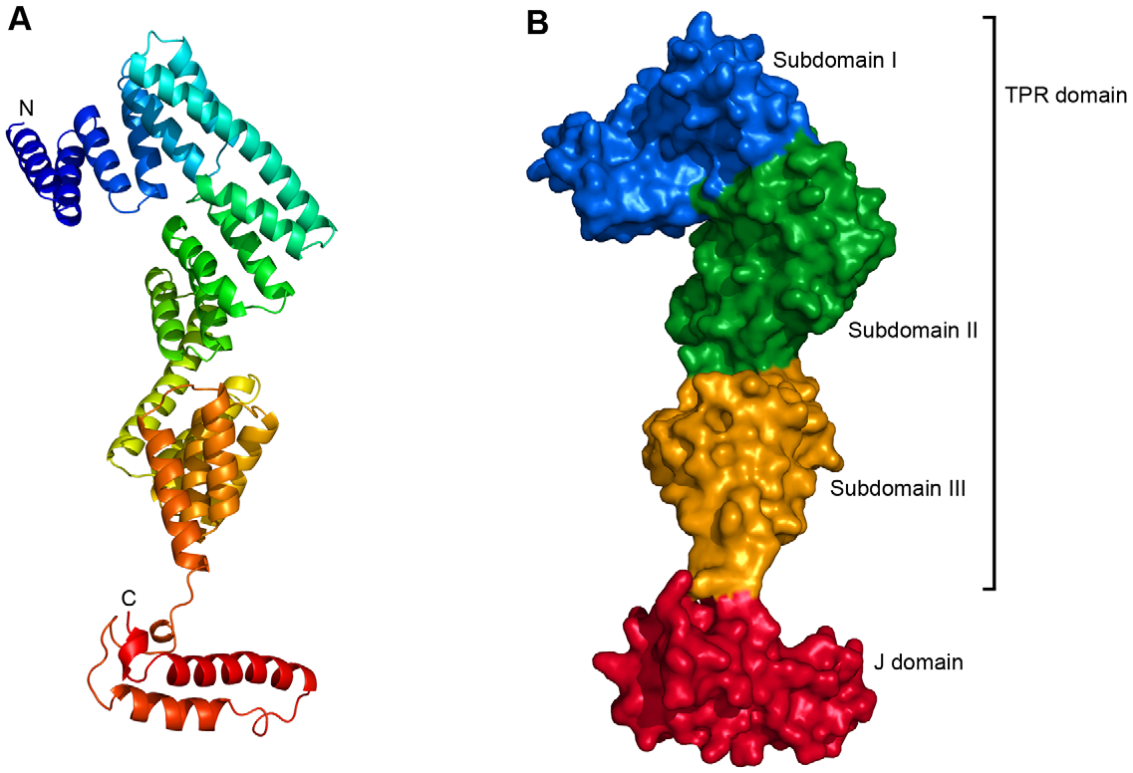
Overall structure of human P58^IPK^. (A) A cartoon representation of the structure of human P58^IPK^, coloured from blue at the N-terminus to red at the C-terminus. (B) A surface representation of P58^IPK^, with the three TPR subdomains and the J domain depicted in blue, green, yellow and red, respectively.

P58^IPK^ is an entirely α-helical protein with a very elongated monomer structure, approximately 120 Å long but only 20–50 Å wide (Figure 1). The protein used for crystallisation behaves as a monomer in gel-filtration chromatography, in agreement with previously published observations for the TPR domain [9], [12]. PISA [13] suggests the oligomeric state to be monomeric in crystal form 2, but identifies a potential trimer, generated by the crystallographic symmetry, in crystal form 1. However, no previous data support such a trimeric quarternary structure and in light of the gel-filtration data it does not seem likely to be physiologically relevant. The N-terminal TPR domain (residues 35–392) consists of 19 α helices arranged in nine TPR motifs, motifs of 34 amino acid residues which adopt a helix-turn-helix arrangement and often pack together to form domains mediating protein-protein interactions and the assembly of multiprotein complexes [14], [15]. In P58^IPK^ this part of the protein is further divided into three subdomains, each containing three TPR motifs. Subdomain I is composed of helices 1–7 and TPR1-TPR3, subdomain II is composed of helices 7–13 and TPR4-TPR6, and subdomain III is composed of helices 13–19 and TPR7-TPR9. Although the sequence identity between the TPR subdomains is low, the folds of the three subdomains are similar.

The J domain is composed of residues 393–455. The first three residues of the J domain (393–395) adopt an extended conformation, and are followed by a very short helical segment and a long loop (residues 398–408). This section, which essentially forms a linker between the TPR domain and the main body of the J domain, is then followed by two longer anti-parallel helices (residues 408–421 and 429–448, respectively). After the second long helix the density gets weaker, indicating flexibility, although residues 452–454 form another single turn of helix. The last six residues of the J domain are not visible in the electron density, despite being present in the crystallised protein. This, together with our observation that protein constructs including the C-terminal amino acid residues are not soluble, would appear to confirm predictions that the extreme C-terminus of P58^IPK^ (approximately residues 455–504) is unstructured.

There are two disulfide bonds in the structures of P58^IPK^; one between Cys248 and Cys258 linking helices 12 and 13 and one between Cys313 and Cys329 linking helices 15 and 16. This is consistent with P58^IPK^'s primary location in the oxidative environment of the ER. Both disulfides are located in the core of the protein.

### Comparison to the TPR domain of the mouse homologue

**Figure 2:**
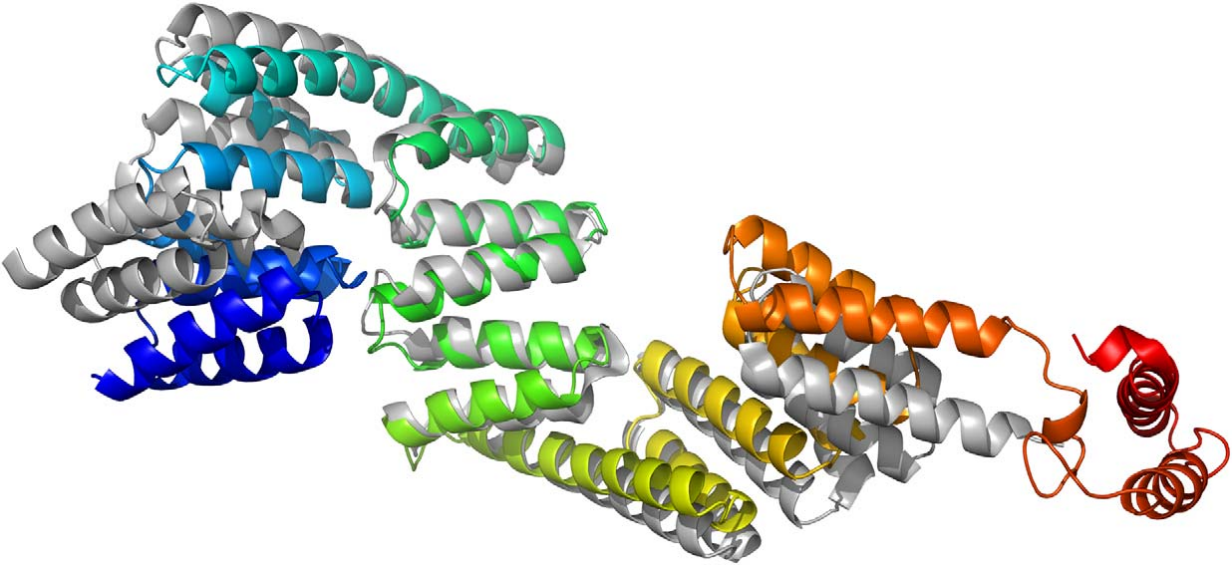
Comparison between human and mouse P58^IPK^ TPR domains. A superimposition of the human P58^IPK^ structure and the previously published structure of the TPR domain of mouse P58^IPK^. Human P58^IPK^ is coloured from blue at the N-terminus to red at the C-terminus, the TPR domain of mouse P58^IPK^ in grey. The structural alignment was based on TPR subdomain II.

A partial structure of mouse P58^IPK^ was recently published, containing only the TPR domain (PDB ID: 3IEG) [9]. Despite very high sequence identity between the two homologues, the overall structure of the human TPR domain has a significantly different shape than that of the mouse protein. Superimposing amino acid residues 35–393 gives an RMSD of 3.7 Å (Figure 2). This difference is caused primarily by rotations of the long helices 7 and 13 connecting subdomain II to subdomains I and III, respectively, which bring all three subdomains closer together and give the human structure a more curved shape.

When solving the human P58^IPK^ structure, we found that using the complete mouse TPR domain as search model for molecular replacement was unsuccessful; a solution could only be found after removal of the N-terminal half of the mouse structure from the model. The differences between the human and mouse crystal structures explain this observation, but also raise the question of whether the observed differences have any physiological relevance. It is possible that there could in fact be a structural difference between the two homologues, although this is unlikely considering their very high sequence identity. The isolated context of the mouse TPR domain could also account for the differences between the two structures; the absence of the J domain could potentially affect the shape of the TPR domain. A third, and perhaps more likely, possibility is that the differences arise as an effect of crystal packing. Each chain in our structure makes similar crystal packing interactions, and these are indeed different from those seen in the crystals of the mouse TPR domain. However, regardless of the reason, the observed differences in structure do emphasise an inherent flexibility of P58^IPK^, which is very likely to be important for its function. P58^IPK^ binds multiple interaction partners, often two or more simultaneously, and the flexibility is likely to facilitate this.

### J domain and BiP binding site

**Figure 3:**
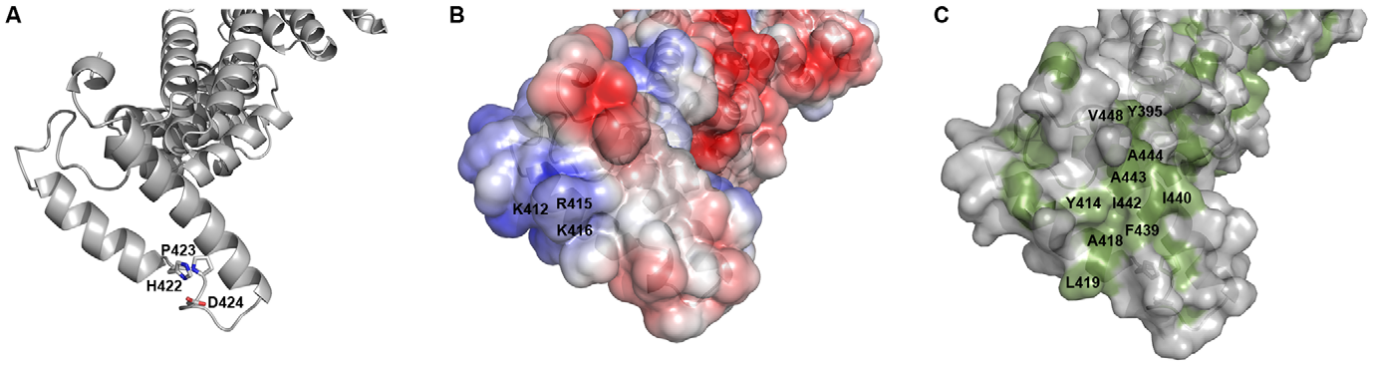
The J domain of P58^IPK^. (A) A cartoon representation of the J domain of P58^IPK^. The residues of the HPD motif are shown in stick representation and coloured by atom type with carbon in grey, oxygen in red, and nitrogen in blue. (B) A surface representation of the J domain, showing the electrostatic surface potential with positively charged regions in blue and negatively charged regions in red. Positively charged residues on helix II are labelled. (C) A surface representation of the J domain, with hydrophobic residues in green. Residues contributing to the hydrophobic patch adjacent to the HPD motif are labelled.

The J domain is the common feature of all proteins in the Hsp40 family; type I and type II Hsp40s contain additional conserved domains, while the type III Hsp40-like proteins, such as P58^IPK^, share only the J domain. The structures of J domains from several different Hsp40s from various organisms have been solved, and they all contain four alpha helices; one short (I), two longer (II and III) and another short (IV). Between the second and third helices lies a conserved histidine-proline-aspartate (HPD) motif which is required for Hsp70 interaction [16]. Our structures provide the first structural data on the J domain of P58^IPK^, and confirm the fold to be similar to those of other Hsp40s. A Dali [17] search using only the J domain reveals the closest structural homologue to be HscB from *Vibrio cholerae* (PDB ID: 3HHO), which aligns with an RMSD of 1.5 Å over 61 amino acid residues. The structure of the two long helices, and the loop between them, is very similar to previous J domain structures, but in P58^IPK^ the two short helices are barely present, with each represented by just one turn of a helix (Figure 3A). In the case of helix I, this is part of the linker connecting the TPR domain and the J domain, and a shorter helix could contribute to the flexibility of the protein. Since the protein is known to function primarily by interactions with other proteins, such as in its proposed role in passing an unfolded protein to the chaperone BiP, it is conceivable that a degree of flexibility of the protein could be beneficial to its function. Helix IV is shorter in P58^IPK^ than in previously published J domains, and the subsequent residues are disordered and not seen in the structure, but it is possible that the residues missing from the extreme C-terminus, although predicted themselves to be disordered, could influence the structure here.

Previous studies have confirmed that the conserved HPD motif of P58^IPK^ is important for its specific interaction with Hsp70 proteins, including BiP; point mutations introduced into the motif resulted in the loss of J domain function, consistent with other Hsp40-Hsp70 interactions [18], [19]. The P58^IPK^ construct used for crystallisation includes the complete J domain, and the structure allows us to examine the area around the HPD motif in detail for the first time. The structure confirms that this HPD motif (residues 422–424), and therefore the predicted BiP-interaction site, is located at the start of the loop between the two large helices of the J domain (helix II and helix III). The motif is positioned at the very edge of the elongated P58^IPK^ structure, allowing plenty of space for interaction with the large BiP protein, and the sidechains of both the aspartate and the histidine are on the surface of the protein.

Significant evidence accumulated from structural, mutagenesis and peptide-inhibition studies has shown that the Hsp40-Hsp70 interaction requires helix II of a J domain, the loop between the second and third helices and, potentially to a lesser extent, helix III [20]–[23]. Positively charged residues of helix II have been particularly strongly implicated, and are thought to interact with negatively charged residues in the binding cleft of the Hsp70 nucleotide binding domain (NBD) [21], [24]. In P58^IPK^ the region encompassing the surface of helix II, the loop and the start of helix III is indeed comprised of a combination of positively charged and hydrophobic residues, and the charged residues are localised primarily to helix II, in agreement with previous data. However, P58^IPK^ has a lower proportion of charged surface in this area than most other J domain structures, and those residues that are present (Lys412, Arg415, and Lys416) are on the N-terminal end of helix II which is furthest from the HPD motif (Figure 3B). Instead the J domain of P58^IPK^ has a higher proportion of hydrophobic surface; immediately adjacent to the HPD motif is situated a patch of hydrophobic residues. This hydrophobic patch, which includes residues Tyr395, Tyr414, Ala418, Leu419, Phe439, Ile440, Ile442, Ala443, Ala444, and Val448, has approximate dimensions of 12 by 15 Å (Figure 3C). In other J domain structures there is frequently a hydrophobic patch next to the HPD motif, but its size and position are not consistent; that seen in the human P58^IPK^ structure is amongst the more extensive. Given its position, it is reasonable to speculate that this hydrophobic patch could be involved in the interaction between P58^IPK^ and BiP, and that the interaction might therefore be more of a hydrophobic, rather than charged, nature. While the exact binding site on BiP is not known, there is a conserved arginine residue in the Hsp70 family which has been previously shown in several cases to interact with the HPD motif of J domains [22], [25]. The equivalent residue in BiP, Arg197, which has already been implicated in the interaction with two other mammalian ERdj proteins [26]–[28], is surrounded by hydrophobic surface residues in the structure of human BiP. If this residue does also interact with the J domain of P58^IPK^, the BiP structure would therefore be entirely consistent with our hypothesis.

**Figure 4:**
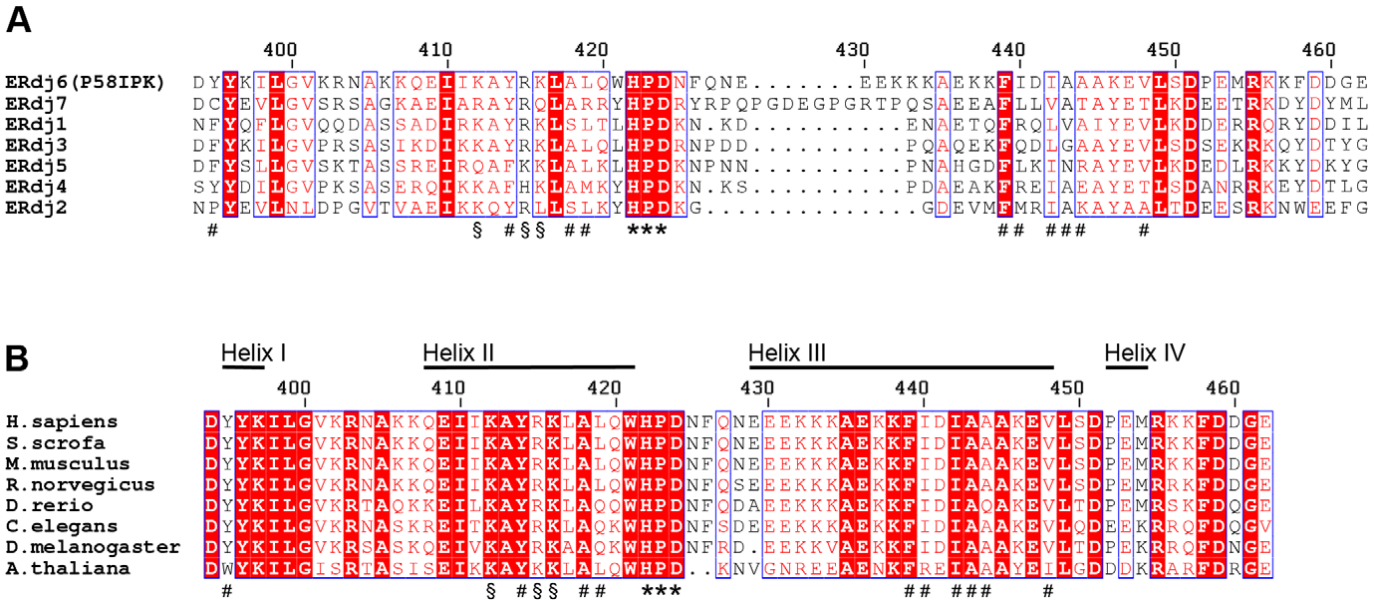
Sequence conservation between J domains from the human ERdj proteins and from P58^IPK^ from different species. (A) Alignment of sequences of the J domains from the human ERdj proteins. The surface residues mentioned in the text are labelled, using (*) for the residues of the conserved HPD motif, (#) for residues of the adjacent hydrophobic patch in human P58^IPK^, and (§) for positively charged residues on helix II. Conserved residues are shown with red background, and residue numbering corresponds to human P58^IPK^. (B) Sequence alignment of the J domains from P58^IPK^ from different species. Labelling is the same as in (A).

Interestingly, the residues surrounding the HPD motif do not all coincide with those that are best conserved within the human BiP-binding ERdj family. Each of the positively charged residues on helix II is conserved in five out of the seven proteins but of the residues in the hydrophobic patch, only those closest to the HPD motif are highly conserved; with Phe439 present in all of the seven ERdj proteins, and Tyr414 and Ala418 present in five (Figure 4A). This is not completely unexpected; as the interaction partner, human BiP, is the same for each, it follows that different interaction characteristics must be conferred by differences in the J domains. In contrast, the residues surrounding the HPD motif, as well as the J domains in general, are very highly conserved between P58^IPK^ homologues from various organisms (Figure 4B). Mutagenesis experiments are underway in order to determine which of these surface residues are directly involved in the P58^IPK^-BiP interaction.

### Potential binding sites for other interaction partners

**Figure 5:**
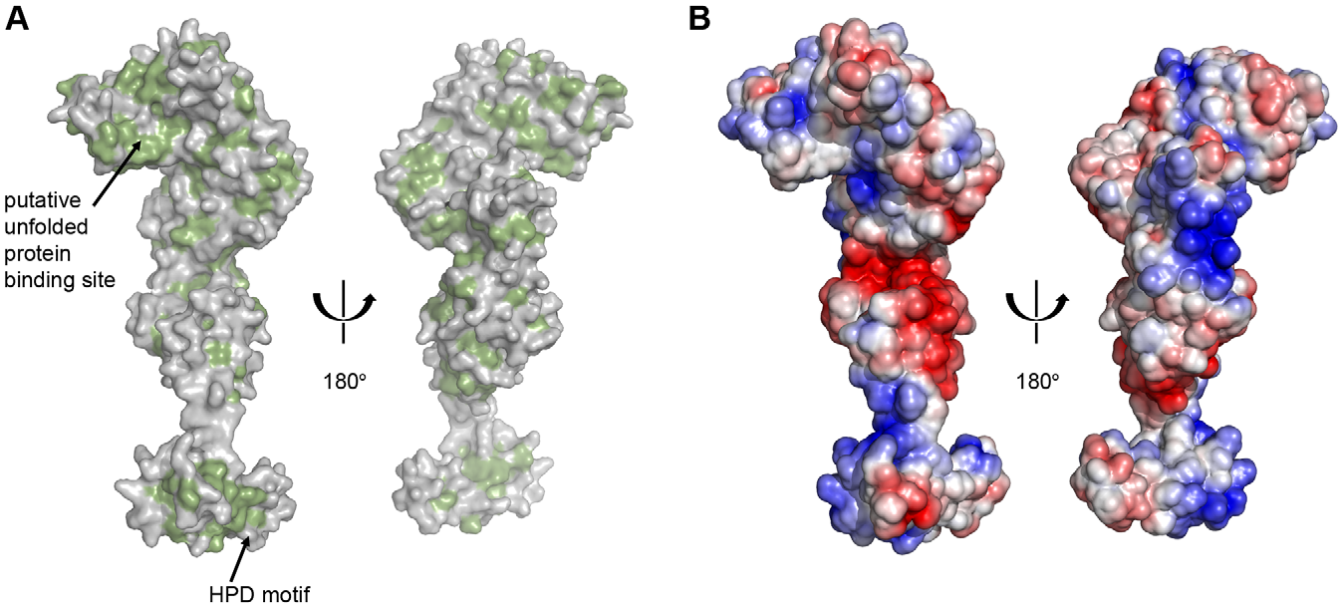
The surface of P58^IPK^. (A) Surface representations of human P58^IPK^, with hydrophobic residues highlighted in green. (B) Electrostatic surface representations with positively charged regions in blue and negatively charged regions in red.

It is known that the varied functions of P58^IPK^ in the cell require binding of several very different interaction partners, and it is therefore of particular interest to analyse the remainder of the surface of the protein. The solvent-accessible surface of P58^IPK^ is approximately 24400 Å^2^. A hydrophobic surface analysis (Figure 5A) shows a major patch of hydrophobic residues lining a groove close to the N-terminus of the protein. This hydrophobic patch, which includes residues Leu48, Ala49, Ala50 Leu53, Leu57, Phe60, Tyr71, Ile72, Tyr74, Tyr75, Ala78, Phe81, Leu82, Ala83, Met84, Leu94, Leu100, Phe104, Ala106, Leu109 and Leu114, is approximately 20 by 25 Å, covering a large portion of the surface of TPR subdomain I. This is the same hydrophobic patch that was also identified in mouse P58^IPK^ [9]. Despite the obvious differences in overall conformation between the structure of human P58^IPK^ and the previous structure of the mouse TPR domain, both the shape and the position of the patch are highly conserved. This would be consistent with an important role in binding unfolded proteins, as suggested by the authors of the mouse TPR domain structure. The same authors also suggested that P58^IPK^ interacts with unfolded proteins via its TPR domain, recruits BiP via the J domain and then delivers the substrate to BiP. The structure of human P58^IPK^ certainly does not rule out this theory in any way, but does raise one interesting point. P58^IPK^ is a particularly elongated protein and the distance between the putative unfolded peptide binding site in TPR subdomain I and the BiP binding site is therefore very large, at approximately 100 Å. The mechanism by which P58^IPK^ would transfer an unfolded protein substrate to BiP is not yet understood, but given the conformational difference already seen between the TPR domain in our structure and that of the mouse homologue, it is possible that P58^IPK^ in the complex could undergo a structural rearrangement to bring the two sites closer together. It has also previously been suggested that some Hsp40s could interact additionally with other regions of their Hsp70 partner, beyond the classical J domain – NBD interaction [29]. Taking into account the distance between the J domain and the predicted binding site for unfolded protein this could be envisaged as a possibility in this case, however previous studies have indicated that a P58^IPK^ construct without the J domain does not interact with BiP [8].

While the BiP-interaction site can be localised by the conserved HPD motif, the exact binding sites of P58^IPK^'s other interaction partners are more difficult to predict from current data. The interaction between P58^IPK^ and PKR, which inhibits dimerisation of the kinase [30], has been shown to involve TPR6 [31], [32]. However, the detailed mechanism of inhibition and the exact residues of P58^IPK^ that mediate the interaction remain to be defined, and surface analysis of TPR6 in our structure does not reveal any obvious binding sites. The cytosolic regulatory protein P52^rIPK^ has been proposed to bind to and inhibit P58^IPK^ at TPR7 [33]. The surface of TPR7 again does not show any extensive hydrophobic patches, but this part of the protein has a high proportion of charged surface (Figure 5B). It has been shown that P52^rIPK^ binds to P58^IPK^ via a charged domain that has limited homology to the charged domain of Hsp90 [33], and the charged surface of TPR7 suggests that this interaction could be of an electrostatic nature. Further experiments will be required in order to test this hypothesis and indeed to fully characterise the interactions between P58^IPK^ and its diverse interaction partners. However this structure, the first structure of P58^IPK^ with the J domain, has provided new insights into the protein, and will now provide the basis for detailed interaction studies and other experiments to further characterise the functions of P58^IPK^ in the cell and how these functions are brought about by its many interactions with other proteins.

## Materials and Methods

### Cloning

The cDNA corresponding to the TPR repeats and the J domain (residues 35–461) of human P58^IPK^ was amplified by the polymerase chain reaction and cloned into pNIC28-Bsa4 by ligation-independent cloning [34]. The resulting expression construct contained a hexahistidine tag and a TEV-protease cleavage site (MHHHHHHSSGVDLGTENLYFQSM) at the N-terminus. The sequences of the cloned constructs were verified by sequence analysis.

### Expression and purification

Recombinant human P58^IPK^ was expressed in *Escherichia coli* Rosetta (DE3) cells. Cultures in LB medium were incubated with shaking at 37°C to OD_600_ 0.5–0.8, at which point the temperature was lowered to 18°C and recombinant protein production was induced by addition of isopropyl β-D-thiogalactopyranoside to a final concentration of 0.2 mM. The cultures were then grown for an additional 17–21 h at 18°C before harvest by centrifugation. Cell pellets were frozen and stored at −20°C until further use. Selenomethionine-substituted protein was produced by suppression of methionine biosynthesis using standard methods [35].

Prior to purification, the cell pellets were thawed and resuspended in 5 ml/g cell weight of lysis buffer (50 mM sodium phosphate, pH 8.0, 500 mM NaCl, 5 mM imidazole) supplied with Complete protease inhibitor cocktail (Roche) and lysed by sonication at 4°C. Cell debris was removed by centrifugation and the soluble fraction was filtered through double syringe filters (0.45 µm and 0.22 µm pore size). The protein was purified in three consecutive steps of chromatography. The cell lysate was loaded onto a HisTrap HP column (GE Healthcare) and eluted with increasing imidazole concentration, followed by anion exchange on a Q HiTrap column (GE Healthcare) against increasing concentration of NaCl. Size-exclusion chromatography was performed on a Superdex 200 column (GE Healthcare) using a gel-filtration buffer consisting of 50 mM Tris-HCl, pH 8.0, and 300 mM NaCl. When appropriate, the hexahistidine tag was removed by overnight incubation with TEV protease and separated from P58^IPK^ by Ni-NTA affinity chromatography. Samples containing native and selenomethionine-derivative P58^IPK^ were dialysed against crystallisation buffer (25 mM Tris, pH 8.0, 100 mM NaCl, 2 mM DTT), concentrated to 31 and 18 mg/ml, respectively, and used in subsequent crystallisation experiments. The size of the protein and the incorporation of selenomethionine were verified by mass spectrometry.

### Crystallisation

Initial crystallisation screening was performed by the sitting drop vapour-diffusion method using standard crystal screens (Qiagen, Hampton Research, Emerald Biostructures). Suitable crystallisation conditions were established and optimised for native and selenomethionine-substituted protein. P58^IPK^ was crystallised in two different crystal forms. Crystal form 1 (selenomethionine) grew at 4°C in drops comprised of 1 µl of well solution and 2 µl of protein solution (18 mg/ml). The well solution contained 0.1 M Tris, pH 8.5, 1.3 M succinic acid and 1% (w/v) PEG MME 2000. Crystal form 2 (native, without His-tag) grew at 20°C in drops comprised of 1 µl of well solution and 2 µl of protein solution (31 mg/ml). The well solution contained 0.1 M HEPES, pH 8.2, 1.4 M succinic acid and 1% (w/v) PEG MME 2000. Crystals typically began to grow after 1–2 weeks. Prior to data collection, crystals were cryoprotected using artificial mother liquor containing 25% glycerol and were flash-frozen in liquid nitrogen.

### Data collection and processing

X-ray diffraction data for native and selenomethionine-substituted P58^IPK^ crystals were collected at the European Synchrotron Radiation Facility, Grenoble, France. Anomalous data were collected from a single selenomethionine-substituted crystal of crystal form 1 to 3.2 Å resolution on beamline ID23-1, which is equipped with an ADSC Q315R CCD detector. 120 degrees of data were collected at a wavelength of 0.979 Å using a 1° oscillation angle. Higher resolution native data were collected to a resolution of 3.0 Å on beamline ID14-1 which is equipped with a ADSC Q210 CCD detector, using a single crystal of crystal form 2. 73.15 degrees of data were collected at a wavelength of 0.933 Å using a 0.55° oscillation angle. Data were processed using iMosflm [36] and SCALA from the CCP4 suite [37]. Table 1 shows a summary of the statistics for each of the datasets.

### Phasing, model building and refinement

Phases for crystal form 1 were obtained by a combination of single wavelength anomalous diffraction and molecular replacement (MR-SAD) using the PHENIX [38] interface to Phaser [39], with the C-terminal half (residues 203–393) of the TPR domain of mouse P58^IPK^ (PDB ID: 3IEG) [9] as the search model. SAD phasing alone was sufficient to identify seven selenomethionine sites with a figure of merit (FOM) of 0.53, however MR-SAD was ultimately used for phasing as it gave significantly better preliminary electron density maps and facilitated model building. Electron density maps were improved by density modification in Parrot [40] and RESOLVE [41]. The N-terminal part of the TPR domain was built by manually positioning helices using the mouse structure as a template where possible. After initial refinement of the TPR domain, the J domain was built manually using a high-resolution crystal structure of the J domain of the DnaJ homologue dnj-2 from *C.elegans* (PDB ID: 2QSA) as a template. The structure was refined with alternating cycles of restrained refinement in REFMAC5 [42] v5.5 and manual inspection and model building in COOT [43]. In the later stages of refinement, atomic displacement parameters were refined in REFMAC by the TLS (translation, libration, and screw) method [44]. The structure of the higher resolution crystal form 2 was determined by molecular replacement in Phaser, using the structure from crystal form 1 as the search model. Three molecules were found in the asymmetric unit. Iterative rounds of model building and refinement were carried out using COOT and REFMAC5, respectively. Initial refinement was performed in REFMAC5 v5.5 with medium NCS restraints and overall B-factor refinement. Later refinement was performed in REFMAC5 v5.6 with isotropic B-factor refinement. Atomic displacement parameters were refined in the final stages by the TLS method with each of the three monomers in the asymmetric unit treated as a single TLS group. The geometry of each model was checked using the validation functions of COOT in addition to final analysis in MolProbity [45], and the fit between model and data was assessed in SFCHECK [46]. The results from refinement are summarised in Table 2. Structure alignments were carried out using the SSM superposition [47] function in COOT. Calculations of accessible surface area were performed using the PISA server [13]. The figures were prepared in PyMOL [48]. Composite omit maps were calculated in CNS [49].

### Accession numbers

Structure factors and the coordinates of the final models have been deposited in the Protein Data Bank (http://www.rcsb.org) with accession numbers 2Y4T and 2Y4U.

## Supporting Information

Figure S1 **Composite omit maps.** Simulated annealing composite omit maps contoured at 1.0 σ showing the electron density quality in different parts of chain A in the 3.0 Å resolution structure. (A) The TPR domain. (B) The J domain. (C) The linker between the TPR domain and the J domain.
(TIF)
