# Supplementary figures and images for: The Crystal Structure of the Human Co-Chaperone P58IPK
Source: PLoS One. 2011 Jul 25;6(7):e22337. doi: 10.1371/journal.pone.0022337 (PMC3143134; doi:10.1371/journal.pone.0022337)

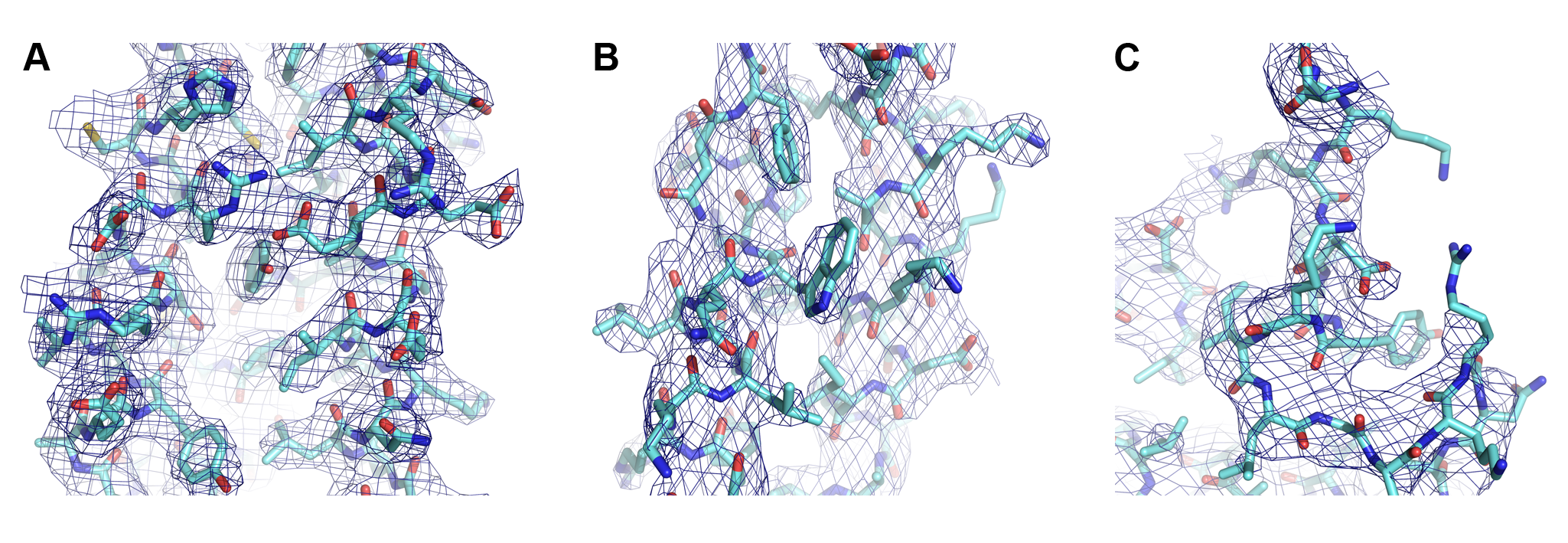

Supplement: Figure S1 — Composite omit maps. Simulated annealing composite omit maps contoured at 1.0 σ showing the electron density quality in different parts of chain A in the 3.0 Å resolution structure. (A) The TPR domain. (B) The J domain. (C) The linker between the TPR domain and the J domain. (TIF) [file pone.0022337.s001.tif]
